# Diversity of Porifera in the Mediterranean coralligenous accretions, with description of a new species

**DOI:** 10.3897/zookeys.336.5139

**Published:** 2013-09-27

**Authors:** Marco Bertolino, Carlo Cerrano, Giorgio Bavestrello, Mirco Carella, Maurizio Pansini, Barbara Calcinai

**Affiliations:** 1 Dipartimento di Scienze della Terra dell’Ambiente e della Vita (Di.S.T.A.V.), Università di Genova, Corso Europa, 26–16132 Genova, Italy; 2 Dipartimento di Scienze della Vita e dell’Ambiente (Di.S.V.A.), Università Politecnica delle Marche, Via Brecce Bianche–60131 Ancona, Italy

**Keywords:** Porifera, cryptic species, bioconstructions, Ligurian Sea

## Abstract

Temperate reefs, built by multilayers of encrusting algae accumulated during hundreds to thousands of years, represent one of the most important habitats of the Mediterranean Sea. These bioconstructions are known as “coralligenous” and their spatial complexity allows the formation of heterogeneous microhabitats offering opportunities for a large number of small cryptic species hardly ever considered.

Although sponges are the dominant animal taxon in the coralligenous rims with both insinuating and perforating species, this group is until now poorly known. Aim of this work is to develop a reference baseline about the taxonomic knowledge of sponges and, considering their high level of phenotypic plasticity, evaluate the importance of coralligenous accretions as a pocket for biodiversity conservation.

Collecting samples in four sites along the coast of the Ligurian Sea, we recorded 133 sponge taxa (115 of them identified at species level and 18 at genus level). One species, *Eurypon gracilis* is new for science; three species, *Paratimea oxeata*, *Clathria (Microciona) haplotoxa* and *Eurypon denisae* are new records for the Italian sponge fauna, eleven species are new findings for the Ligurian Sea. Moreover, seventeen species have not been recorded before from the coralligenous community. The obtained data, together with an extensive review of the existing literature, increase to 273 the number of sponge species associated with the coralligenous concretions and confirm that this habitat is an extraordinary reservoir of biodiversity still largely unexplored, not only taxonomically, but also as to peculiar adaptations and life histories.

## Introduction

The term “coralligenous” refers to a secondary hard substrate, formed by the concretion of algal thalli and, to a lesser extent, by animal skeletons. Two main types of coralligenous concretions can be distinguished: banks, which are built over more or less horizontal substrata, and rims, which develop in the outer parts of marine caves and on vertical cliffs (Ballesteros 2006). Coralligenous communities represent the temperate reefs of the Mediterranean Sea and along with the meadows of *Posidonia oceanica* (Boudouresque, 2004) are biodiversity hot spots in the basin. The holes and crevices of the coralligenous build-ups support a complex community dominated by suspension feeders (sponges, hydrozoans, serpulid polychates, molluscs, bryozoans and tunicates).

Laubier (1966) first emphasized the high biodiversity of the coralligenous and listed 544 invertebrate species from this assemblage in Banyuls. Later, Hong (1980), in an exhaustive survey of the coralligenous of Marseille, listed a total of 682 species, whilst other authors (Ros et al. 1984) reported 497 species of invertebrates from the algal concretions of the Medes Islands. Recently, Romdhane (2003) reported 35 algal species and 93 animal species from a coralligenous formation along a vertical cliff in the gulf of Tunis. However, the number of species living in the coralligenous assemblages is still undefined, because of the richness of the fauna (Laubier 1966), the habitat complexity (Pérès and Picard 1964, Ros et al. 1985), the wide depth range of the conglomerates (Ballesteros 2006), the sporadic presence of cryptic species and the scarcity of reference studies. A rapid, non-destructive protocol for biodiversity assessment and monitoring of coralligenous, based on photographic sampling, was recently proposed by Kipson et al. (2011).

Sponges, with 142 recorded species, are one of the most diverse group of sessile animals of the coralligenous assemblage (Ballesteros 2006). Some species, mainly belonging to the family Clionaidae, are active bioeroders representing the principal driving force in the turn-over of bioconstructions, both in temperate and tropical areas (Cerrano et al. 2001, Calcinai et al. 2000, 2005, 2007c)

In the present paper, the species diversity of the coralligenous sponge fauna was studied in four sites of the Ligurian Sea, focusing on the relatively poorly known cryptic species boring or insinuating into the calcareous concretions. A new species for science and ten poorly known species, rarely recorded in the Mediterranean Sea, are treated exhaustively.

## Materials and methods

**Figure 1.**
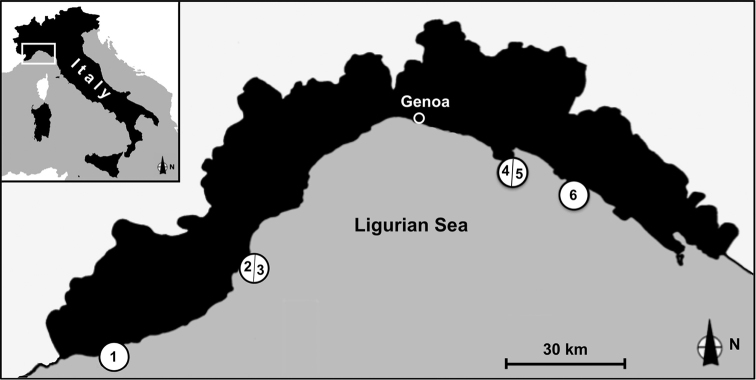
The four studied localities along the Ligurian Coast: Santo Stefano Shoal (station **1**), Gallinara Island (station **2–3**), Punta del Faro (Portofino Promontory) (station **4–5**) and Punta Manara (station **6**).

Samples were collected between 30 and 40 m depth by SCUBA diving from 6 stations along the Ligurian coast where coralligenous is more developed (Fig. 1). Stations (from West to East) are: Santo Stefano Shoals, station 1; Gallinara Island, station 2 (Falconara) and station 3 (Sciusciaù); Portofino Promontory, Punta del Faro, station 4 and 5 (northern and southern side of the point); Punta Manara, station 6. Four blocks of coralligenous concretion, with an average volume of 20 l, were collected from each station.

All the sponge species settled on the surface of these blocks were sampled and identified.

Two of the four blocks from each station were cut into slices about 2 cm thick and observed by a stereomicroscope to detect the cryptic, generally small, endolithic sponges.

The spicule complement of each sponge specimen was analysed according to Rützler (1978). From 30 measurements for each spicule type, size range, mean and standard deviation (in brackets) were calculated. Dissociated spicules were transferred onto stubs and sputtered with gold for SEM analyses and observed with a Philips XL 20 scanning electron microscope. Whenever possible, skeletal architecture was examined in light and scanning electron microscope (SEM) on hand-cut sections of the ectosome and choanosome. Unfortunately, due to small size and cavity dwelling habit, for most specimens it was impossible to study the skeleton.

We followed the classification given by Hooper and van Soest (2002) and the updated nomenclature reported in the World Porifera Database (van Soest et al. 2013). The geographic distribution of sponges in the Mediterranean Sea was compared with that reported by Pansini and Longo (2003, 2008), considering nine biogeographic areas for the Italian seas.

## Results

During this survey we have recorded 133 sponge taxa (115 of them identified at species level and 18 at genus level). One species is new for science, 17 are new findings for the coralligenous conglomerate, 11 of which for the Ligurian Sea and 3 for the Italian sponge fauna (Table 1). In the following taxonomic part we provide the description of the new species and of ten poorly known ones.

**Figure 2.**
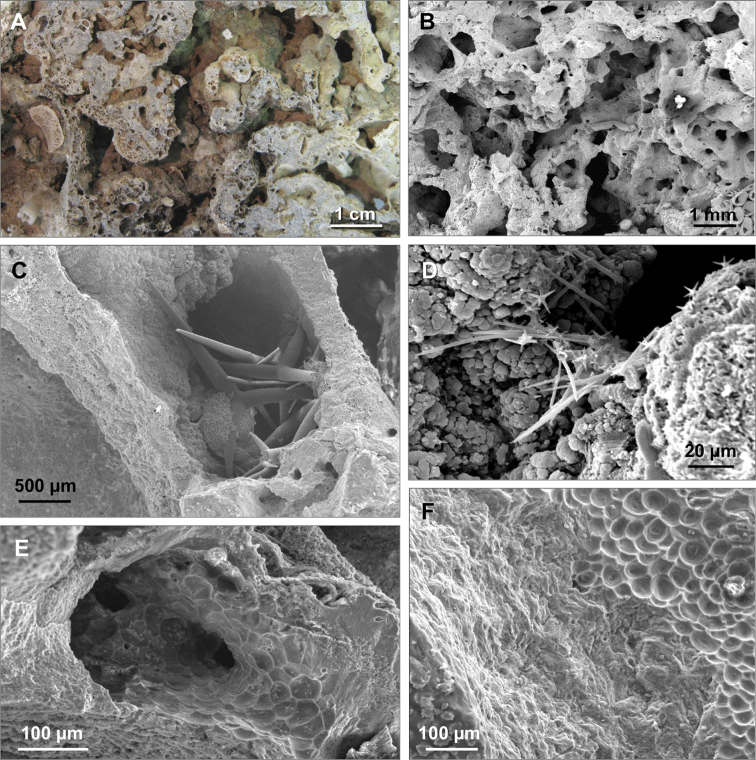
Porosity of the coralligenous concretion. **A** Holes and cavities of the coralligenous concretion **B** Magnification of the holes **C** Magnification of a natural hole occupied by spicules of *Pachastrella monilifera* **D** Spicules of *Jaspis johnstoni* in a natural cavity in the coralligenous concretion **E** Cavity excavated by a boring sponge with excavation marks (pits) on the wall **F** Border between the area excavated by a boring sponge (right) and the not excavated area (left).

On the surfaces of the blocks 103 massive or encrusting species were recorded; inside the crevices of the conglomerate 63 species were observed and 33 shared both positions. Thirty species are exclusively endolithic demonstrating the abundance of cryptic sponges thriving inside the porous matrix of the coralligenous substrate (Table 1) (Fig. 2).

**Table 1.** List of Demospongiae and Homoscleromorpha species living outside and inside the coralligenous blocks (SSS: Santo Stefano Shoals, station 1; GI: Gallinara Island, station 2-3; PF: Punta del Faro, station 4-5; PM: Punta Manara, station 6; * new finding for the coralligenous concretion; ** new finding for the Ligurian Sea; *** new finding for the Italian sponge fauna).

**Species \ Sites**	**SSS**	**GI**	**PF**	**PM**	**Epilithic**	**Endolithic**
*Oscarella lobularis* (Schmidt, 1862)			+	+	+	
*Plakina trilopha* Schulze, 1880	+	+				+
*Plakinastrella copiosa* Schulze, 1880	+					+
*Plakortis simplex* Schulze, 1880			+		+	+
*Samus anonymus* Gray, 1867	+	+				+
*Stelletta grubii* Schmidt, 1862	+					+
*Stelletta lactea* Carter, 1871 *		+				+
*Stelletta stellata* Topsent, 1893 *				+		+
*Jaspis incrustans* Topsent, 1890 **		+	+	+		+
*Jaspis johnstoni* (Schmidt, 1862)	+	+	+	+	+	+
*Penares euastrum* (Schmidt, 1868)	+		+	+	+	+
*Dercitus (Stoeba) plicatus* (Schmidt, 1868)	+	+	+	+	+	+
*Pachastrissa* sp.	+					+
*Erylus discophorus* (Schmidt, 1862)	+			+	+	+
*Geodia conchilega* Schmidt, 1862	+	+	+		+	+
*Geodia cydonium* Schmidt, 1862	+		+		+	+
*Pachastrella monilifera* Schmidt, 1868		+	+			+
*Poecillastra compressa* (Bowerbank, 1866)	+		+		+	+
*Triptolemma simplex* (Sarà, 1959)	+	+	+		+	+
*Cliona burtoni* Topsent, 1932 *^,^ **		+				+
*Cliona celata* Grant, 1826	+		+	+	+	+
*Cliona janitrix* Topsent, 1932	+	+	+	+	+	+
*Cliona schmidtii* (Ridley, 1881)				+	+	+
*Cliona viridis* Schmidt, 1862	+	+		+	+	+
*Cliona* sp.			+		+	+
*Dotona pulchella mediterranea* Rossell & Uriz, 2002	+					+
*Spiroxya corallophila* (Calcinai et al., 2002)			+			+
*Spiroxya heteroclita* Topsent, 1896	+	+	+		+	+
*Spiroxya sarai* Melone, 1965		+	+			+
*Delectona ciconiae* Bavestrello, Calcinai & Sarà, 1996			+			+
*Delectona* sp.		+	+		+	
*Paratimea oxeata* Pulitzer-Finali, 1978 *^,^ **^,^ ***	+					+
*Polymastia* sp.		+	+		+	
*Diplastrella bistellata* (Schmidt, 1862)	+	+	+		+	+
*Aaptos aaptos* (Schmidt, 1864)	+		+		+	+
*Prosuberites longispinus* Topsent, 1893		+				+
*Pseudosuberites sulphureus* (Bowerbank, 1866)			+	+	+	
*Suberites carnosus* (Johnston, 1842)				+	+	
*Suberites domuncula* (Olivi, 1792)			+		+	
*Suberites* sp.	+	+			+	
*Terpios gelatinosa* (Bowerbank, 1866)			+	+	+	
*Timea stellata* (Bowerbank, 1866)		+	+	+	+	+
*Timea unistellata* (Topsent, 1892)	+	+			+	+
*Chondrosia reniformis* Nardo, 1847	+		+	+	+	
*Acarnus souriei* Levi, 1952 *^,^ **			+			+
*Acarnus* sp.			+			+
*Clathria (Microciona) armata* (Bowerbank, 1866) *^,^ **		+			+	
*Clathria (Microciona) atrasanguinea* (Bowerbank, 1862)		+		+	+	
*Clathria (Microciona) gradalis* Topsent, 1925	+				+	
*Clathria (Microciona) haplotoxa* (Topsent, 1928) *^,^ **^,^ ***		+			+	
*Clathria (Microciona) toxistyla* (Sarà, 1959)			+		+	
*Clathria (Microciona) toxivaria* (Sarà, 1959)	+				+	
*Clathria (Microciona)* sp.		+	+			+
*Antho (Antho) involvens* (Schmidt, 1864)			+		+	
*Eurypon* cf. *cinctum* Sarà, 1960		+		+	+	
*Eurypon clavatum* (Bowerbank, 1866)	+	+	+	+	+	
*Eurypon coronula* (Bowerbank, 1874) **		+			+	
*Eurypon denisae* Vacelet, 1969 *^,^ **		+			+	
*Eurypon gracilis* sp. n. Bertolino, Calcinai & Pansini		+		+	+	
*Eurypon major* Sarà & Siribelli, 1960	+	+	+	+	+	
*Eurypon topsenti* Pulitzer-Finali, 1983		+	+		+	
*Eurypon vesciculare* Sarà & Siribelli, 1960	+	+	+	+	+	
*Eurypon* sp.	+	+	+	+	+	
*Raspaciona aculeata* (Johnston, 1842)				+	+	
*Raspaciona* sp.				+	+	
*Forcepia (Leptolabis) brunnea* (Topsent, 1904) **		+	+		+	
*Lissodendoryx (Lissodendoryx) isodictyalis* (Carter, 1882)		+			+	
*Lissodendoryx (Anomodoryx) cavernosa* (Topsent, 1892)	+	+		+	+	+
*Crambe crambe* (Schmidt, 1862)	+	+	+		+	
*Crella (Crella) elegans* (Schmidt, 1862)		+			+	
*Crella (Crella) mollior* Topsent, 1925		+			+	
*Crella (Grayella) pulvinar* (Schmidt, 1868)	+	+	+	+	+	
*Hemimycale columella* (Bowerbank, 1864)	+				+	
*Hymedesmia (Hymedesmia) baculifera* Topsent, 1901 *	+	+				+
*Hymedesmia (Hymedesmia) rissoi* Topsent, 1936	+	+			+	+
*Hymedesmia* sp.		+	+		+	
*Hymedesmia (Stylopus) coriacea* (Fristedt, 1866)	+	+	+		+	
*Phorbas fictitius* Bowerbank, 1866	+	+		+	+	
*Phorbas mercator* (Schmidt, 1868) *		+			+	
*Phorbas lieberkuhni* (Burton, 1930)				+	+	
*Phorbas tenacior* (Topsent, 1925)	+	+	+	+	+	
*Phorbas* sp.		+		+	+	
*Plocamionida ambigua* (Bowerbank, 1866) *	+		+	+	+	+
*Tedania (Tedania) anhelans* (Lieberkühn, 1859)			+		+	
*Mycale (Aegogropila) tunicata* (Schmidt, 1862) *				+	+	
*Mycale (Paresperella) serrulata* Sarà & Siribelli, 1960 **^,^ ***		+				+
*Merlia normani* Kirkpatrick, 1908 *			+			+
*Axinella damicornis* (Esper, 1794)	+	+	+	+	+	
*Axinella polypoides* Schmidt, 1862				+	+	
*Axinella verrucosa* (Esper, 1794)	+		+		+	
*Phakellia* sp.				+	+	
*Bubaris carcisis* Vacelet, 1969	+		+		+	+
*Bubaris vermiculata* (Bowerbank, 1866)				+	+	
*Hymerhabdia oxytrunca* Topsent, 1904			+		+	
*Hymerhabdia typica* Topsent, 1892 *			+		+	
*Hymerhabdia* sp.			+		+	
*Halicnemia geniculata* Sarà, 1958 *^,^ **		+			+	
*Halicnemia patera* Bowerbank, 1864				+	+	
*Acanthella acuta* Schmidt, 1862	+	+	+	+	+	
*Dictyonella incisa* (Schmidt, 1880)	+	+	+	+	+	
*Dictyonella marsilii* (Topsent, 1893)			+		+	
*Dictyonella pelligera* (Schmidt, 1862)		+	+	+	+	
*Dictyonella* sp.		+			+	
*Halichondria (Halichondria) contorta* Sarà, 1961		+	+			+
*Halichondria (Halichondria)* cf. *convolvens* Sarà, 1960				+	+	
*Halichondria (Halichondria) genitrix* Schmidt, 1862		+		+		+
*Halichondria (Halichondria) panicea* Pallas, 1766	+		+			+
*Halichondria* sp.	+		+		+	
*Agelas oroides* Schmidt, 1864	+	+	+		+	
*Dendroxea lenis* (Topsent, 1892)	+		+		+	+
*Haliclona (Gellius) angulata* (Bowerbank, 1866)		+		+	+	+
*Haliclona (Gellius) marismedi* (Pulitzer-Finali, 1978) *^,^ **		+		+	+	+
*Haliclona (Halichoclona) fulva* (Topsent, 1893)	+	+	+	+	+	
*Haliclona (Halichoclona) parietalis* (Topsent, 1893)				+	+	+
*Haliclona (Haliclona)* sp.				+	+	+
*Haliclona (Reniera) cinerea* Grant, 1826				+		+
*Haliclona (Reniera) citrina* (Topsent, 1892)				+	+	+
*Haliclona (Reniera)* sp.		+	+	+	+	
*Haliclona (Soestella) arenata* Griessinger, 1971				+		+
*Haliclona (Soestella) mucosa* (Griessinger, 1971)			+		+	
*Haliclona* sp.				+		+
*Siphonodictyon insidiosum* (Johnson, 1899)	+	+	+	+	+	+
*Petrosia (Petrosia) clavata* (Esper, 1794)	+		+	+	+	
*Petrosia (Petrosia) ficiformis* (Poiret, 1798)	+	+	+	+	+	
*Ircinia variabilis* (Schmidt, 1862)	+	+	+	+	+	+
*Sarcotragus spinosulus* Schmidt, 1862	+	+	+	+	+	+
*Cacospongia mollior* Schmidt, 1862	+					+
*Spongia (Spongia) officinalis* Linnaeus, 1759		+			+	
*Spongia (Spongia) virgultosa* (Schmidt, 1868)	+	+	+	+	+	+
*Dysidea avara* (Schmidt, 1862)	+	+		+	+	
*Dysidea* sp.	+					+
*Pleraplysilla spinifera* (Schulze, 1879)	+		+	+	+	
*Aplysina cavernicola* Vacelet, 1959	+				+	
Total number of species	**61**	**70**	**71**	**61**	**103**	**63**

**Figure 3.**
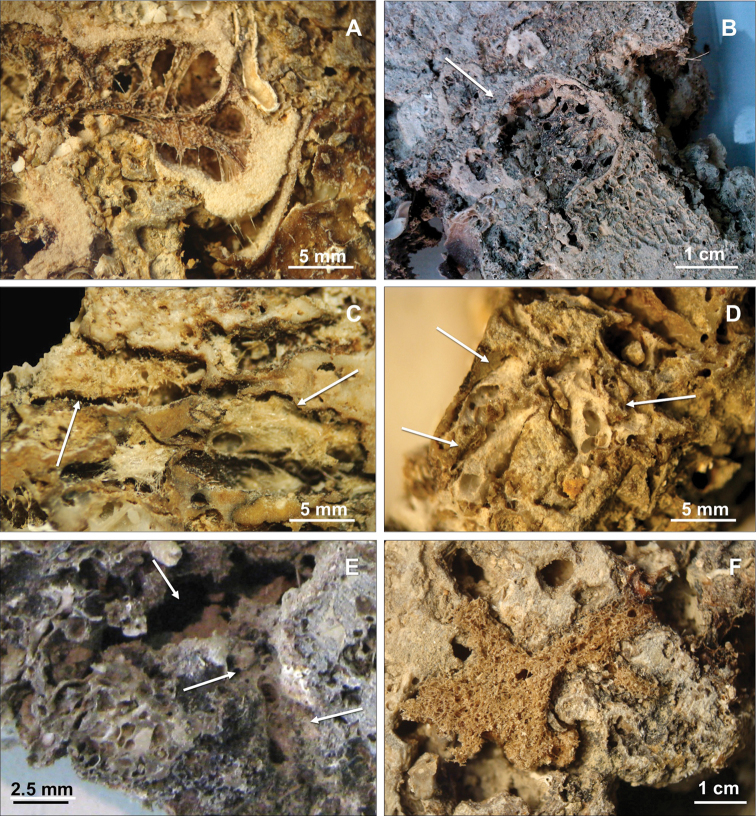
Insinuating sponges. **A** *Geodia cydonium* **B** *Geodia conchilega* **C** *Pachastrella monilifer* a **D** *Poecillastra compressa* **E** *Paratimea oxeata* **F** *Spongia virgultosa*.

Among the 63 species recorded inside the conglomerate, 53 were insinuating and 10 boring (Table 1). From the first group six species: *Geodia cydonium* (Jameson, 1811) (Fig. 3A), *Poecillastra compressa* (Bowerbank, 1866) (Fig. 3D), *Stelletta grubii* Schmidt, 1862, *Paratimea oxeata* Pulitzer-Finali, 1978 (Fig. 3E), *Hymedesmia (Hymedesmia) baculifera* (Topsent, 1901) and *Mycale (Paresperella) serrulata* (Sarà & Siribelli, 1960) were hitherto recorded encrusting or massive; four species: *Erylus discophorus* (Schmidt, 1862), *Penares euastrum* (Schmidt, 1868), *Geodia conchilega* Schmidt, 1862 (Fig. 3B) and *Pachastrella monilifera* Schmidt, 1868 (Fig. 3C) were generally recorded as massive but also described as insinuating by Pulitzer-Finali (1970, 1983) and Calcinai et al. (2007b).

## Species descriptions

### Class Demospongiae
Order Hadromerida
Family Clionaidae
Genus *Cliona*

#### Cliona burtoni

Topsent, 1932

http://species-id.net/wiki/Cliona_burtoni

Figs 4A–L

Cliona burtoni Topsent, 1932: 577.

##### Material examined.

Specimen IG-S-BL1-F5B-spB; dry state, Gallinara Island (station 3, Sciusciaù) 44°01'34"N, 8°13'45"E, depth 30 m, collected 17-06-2009. The specimen was entirely used for spicule preparations.

##### Description.

Boring sponge in alpha growth form, occupying a surface of 1 cm^2^ in a section of conglomerate. Colour beige in dry state.

Skeleton. Not observed.

**Figure 4.**
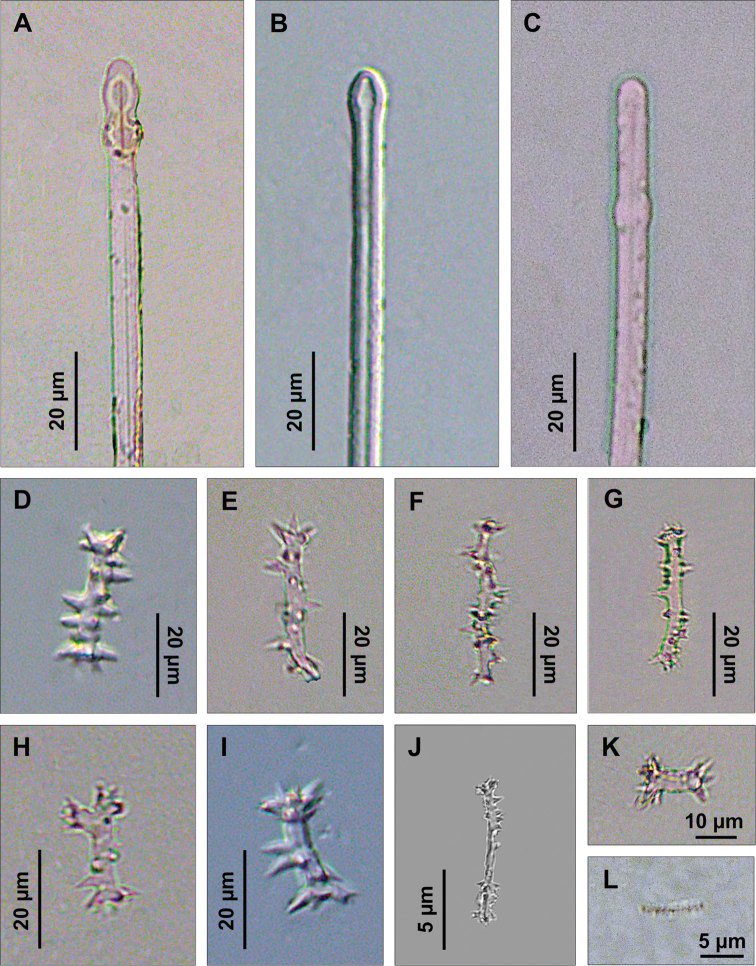
*Cliona burtoni*. **A–C** Tylostyle heads **D–L** Spirasters of various shape and thickness.

Spicules. Macroscleres: tylostyles to subtylostyles straight or slightly curved, 132 (225) 287 × 5 (6) 7.5 μm. Heads with a rounded or oval tyle, sometimes in terminal position but more often shifted along the shaft (Figs 4A, B, C). Microscleres: spirasters of various shape and thickness, straight or curved, 10 (26.5) 45 × 1.25 (10) 17.5 μm. The most abundant have scattered conical spines (Figs 4D, E, F, G, H, I, J, K) and numerous are amphiaster-like (Figs 4H, I, K). The smaller ones are microspinated (Fig. 4J, L).

##### Distribution and discussion.

This is a Mediterranean endemic species (Pansini and Longo 2008) originally described from Corsica (Strait of Bonifacio), where it is known to bore into calcareous rocks and mollusc shells (Topsent 1932). This is a new record for the Ligurian Sea (Gallinara Island) and the coralligenous assemblage and the first finding after the original description.

### Family Hemiasterellidae
Genus *Paratimea*

#### Paratimea oxeata

Pulitzer-Finali, 1978

http://species-id.net/wiki/Paratimea_oxeata

Figs 5A–D

Paratimea oxeata Pulitzer-Finali, 1978: 39.

##### Material examined.

Specimen SSS-BL1-F3A-spH; alcohol and dry state; Santo Stefano Shoals (station 1), 43°49'N, 7°54'E, depth 35 m, collected 14-02-2008. The specimen was entirely used for spicule preparations.

##### Description.

**Figure 5.**
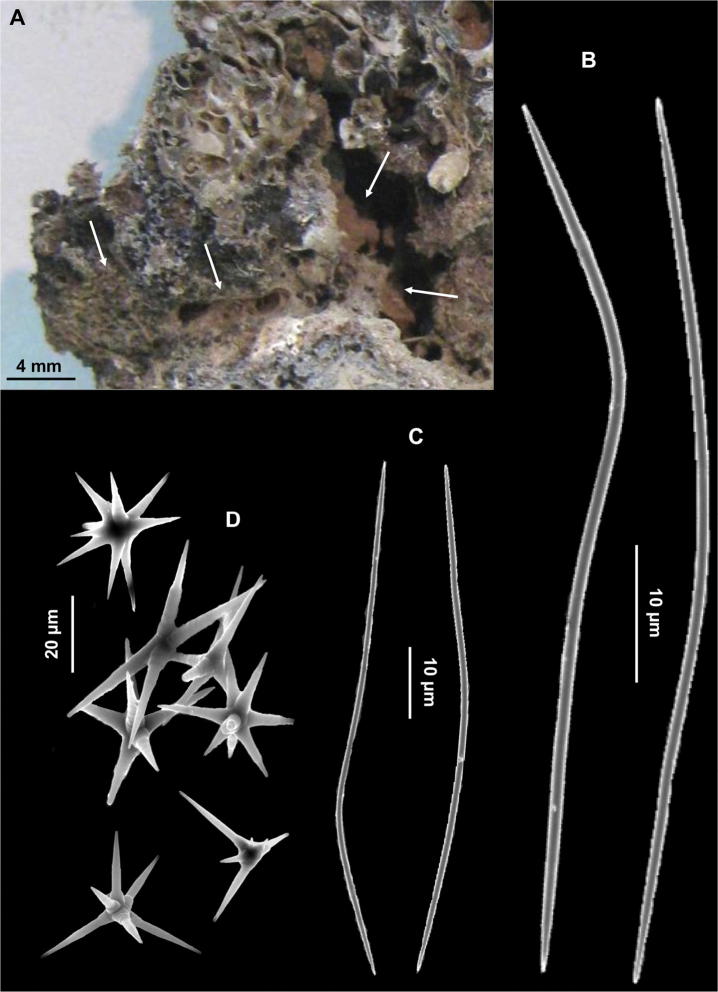
*Paratimea oxeata*. **A** Specimen in the coralligenous accretions (arrows) **B** Large oxeas **C** Small oxeas **D** Oxyasters.

Very small (0.5 cm^2^) insinuating sponge (Fig. 5A) detected inside a cavity of a slice of a coralligenous block. Grey coloured in dry state.

Skeleton. Not observed.

Spicules. Macroscleres: oxeas in two size categories: I) large oxeas curved, bent or flexuous, with hastate tips (Fig. 5B), 810 (961.25) 1200 × 15 (18) 25 μm; II) small oxeas curved or flexuous (Fig. 5C), 300 (546.6) 700 × 2.5 (4.75) 5 μm. Microscleres: oxyasters with more or less marked centrum with 9-12 conical rays, 25 (41.5) 60 μm in diameter. In some cases the number of rays is reduced (Fig. 5D).

##### Distribution and discussion.

The species wasdescribed from Naples (Pulitzer-Finali 1978) where it occurred on rocky bottoms at 60-100 meter depth. This is a new record for the coralligenous assemblage and for the Ligurian Sea and it is probably endemic for the Mediterranean Sea (Pansini and Longo 2008). This is its first finding after the original description.

### Order Poecilosclerida
Suborder Microcionina
Family Microcionidae
Genus *Clathria*
Subgenus *Microciona*

#### Clathria (Microciona) armata

(Bowerbank, 1862)

http://species-id.net/wiki/Clathria_armata

Figs 6A–F

Microciona armata Bowerbank, 1862; 1866: 129.

##### Material examined.

Specimen IG-F-BL4-sp2-fot.; alcohol preserved, Gallinara Island (station 2, Falconara) 44°01'22"N, 8°13'34"E, depth 35 m, collected 31-7-2009.

**Figure 6.**
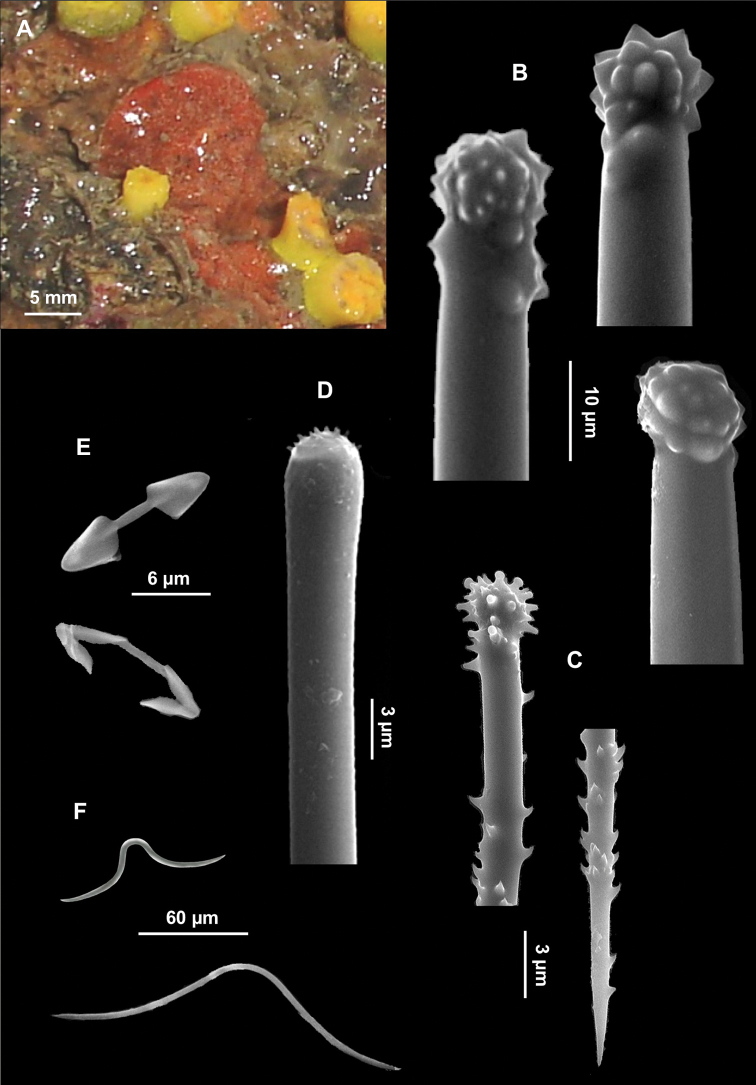
*Clathria (Microciona) armata*. **A** Specimen on the surface of the coralligenous block **B** Large acanthostyle heads **C** Small acanthostyle **D** Subtylostyle with spined head **E** Palmate isochelae **F** Toxas of variable size, with smooth extremities.

**Description.** Thickly encrusting sponge (3-5 mm thick) covering a surface of 1.5 cm^2^ on a coralligenous block (Fig. 6A). Surface irregular, smooth. Consistency soft. The red-orange colour of the living specimen slightly fades when alcohol preserved.

Skeleton. Not observed.

Spicules. Macroscleres: acanthostyles in two size categories: I) large acanthostyles slightly curved, with obtuse spines concentrated on the head (Fig. 6B), 220 (484.5) 830 × 3.75 (8.5) 12 μm; II) small acanthostyles, with scattered spines, but more concentrated on the head (Fig. 6C), 100 (110) 122.5 × 3.75 (5) 6 μm; subtylostyles straight, often with slightly spined head (Fig. 6D), 440 (503.7) 550 × 2.5 (2.9) 3.8 μm. Microscleres: palmate isochelae (Fig. 6E), 10 (12.5) 13.5 μm long. Toxas of variable size, with more or less wide central curvature and slightly reflexed smooth points (Fig. 6F), 80 (114.5) 210 μm long.

##### Distribution and discussion.

This species has been recorded on rocky walls and on mollusc shells from 10 to 180 m depth (Bowerbank 1866, Arndt 1934, Pulitzer-Finali 1983, van Soest and Stone 1986). It is widely distributed in the Mediterranean Sea (Northern Adriatic Sea, Alboran Sea and Ionian Sea (Pansini and Longo 2003, 2008) and along the Atlantic coast of Europe: Arctic, Sweden, Ireland, United Kingdom, France (van Soest et al. 2013).

This specimen, like that described by van Soest and Stone (1986), differs from the type material in the toxa dimensions. Actually Bowerbank measured small toxas 50 µm long and large toxas 130 µm long dividing them in two size categories. Van Soest and Stone (1986) confirm the large variability of spicule size. The species is a new finding for the coralligenous community and the Ligurian Sea.

#### Clathria (Microciona) haplotoxa

(Topsent, 1928)

http://species-id.net/wiki/Clathria_haplotoxa

Figs 7A–F

Leptoclathria haplotoxa Topsent, 1928: 298.

##### Material examined.

Specimen IG-F-BL3-sp5-fot.; alcohol preserved, Gallinara Island (station 2, Falconara) 44°01'22"N, 8°13'34"E, depth 35 m, collected 17-06-2009. The specimen was entirely used for spicule preparations.

##### Description.

**Figure 7.**
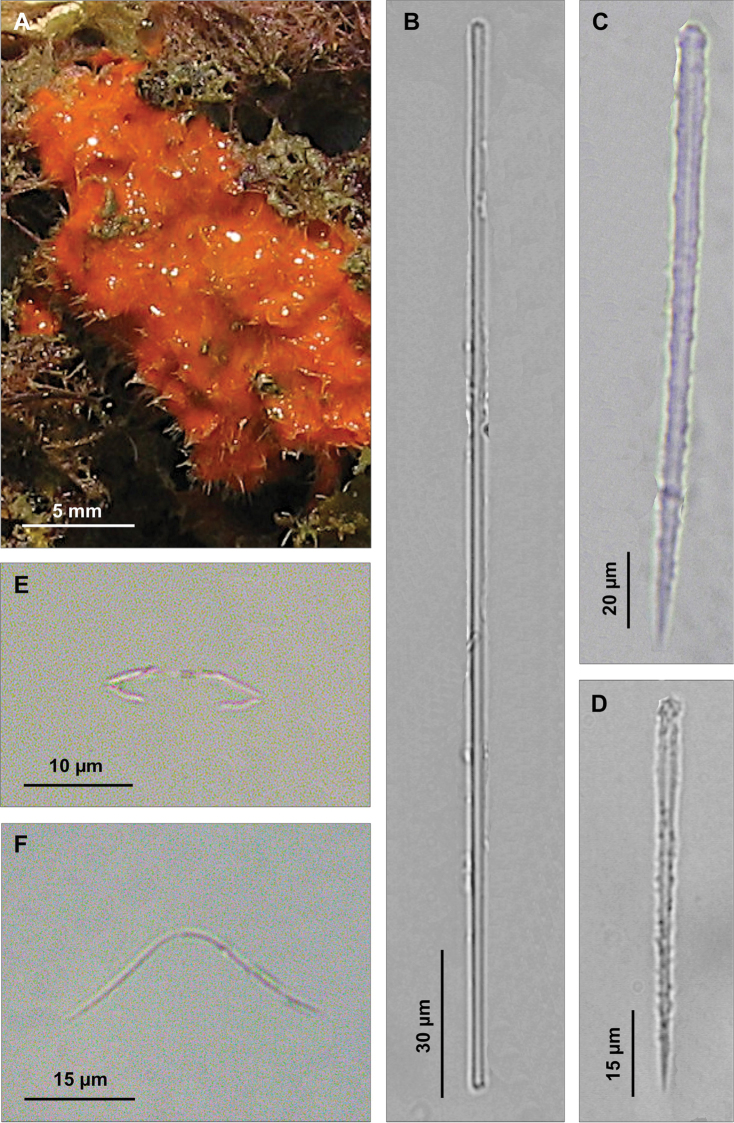
*Clathria (Microciona) haplotoxa*. **A** Specimen on the surface of a coralligenous block **B** Strongyle **C** Large acanthostyle **D** Small acanthostyle **E** Isochela **F** Toxa.

Encrusting sponge on the surface of a coralligenous block, 2 cm in diameter. Surface hispid. Colour brick red (Fig. 7A).

Skeleton. Not observed.

Spicules. Macroscleres: strongyles straight, smooth, 112.5 (178) 215 × 2.5 μm (Fig. 7B); acanthostyles straight with a characteristic constriction under the head, in two size categories: I) large acanthostyles (Fig. 7C), 150 (175.5) 210 μm and II) small acanthostyles (Fig. 7D), 55 (74.5) 102.5 × 2.5 (3.5) 5 μm. Microscleres: palmate isochelae with straight shaft (Fig. 7E), 12.5 (13.8) 15 μm long; toxas thin, smooth, with wide central curvature and slightly reflexed points, 30 (32.5) 37.5 μm long (Fig. 7F).

**Distribution and discussion.** Described from Porto Santo Bay (Madeira) the species extends south to the Sahelian Upwelling (Lévi 1956). In the Mediterranean Sea it was only recorded from Tunisia (Ben Mustapha et al. 2003). It is a new finding for the Italian sponge fauna and for the coralligenous community.

### Family Raspailiidae
Subfamily Raspailiinae
Genus *Eurypon*

#### Eurypon denisae

Vacelet, 1969

http://species-id.net/wiki/Eurypon_denisae

Figs 8A–E

Eurypon denisae Vacelet, 1969: 188.

##### Material examined.

Specimen IG-S-BL3 sp10-fot.; alcohol preserved, Gallinara Island (station 3, Sciusciaù) 44°01'34"N, 8°13'45"E, depth 30 m, collected 31-07-2009.

##### Description.

Encrustingsponge covering a surface of 3 cm^2^ on a coralligenous block. Surface hispid. Colour in life white.

Skeleton. Ectosomal skeleton absent. Choanosomal skeleton consisting of basal acanthostyles with heads embedded in a spongin layer and bundles of very long tylostyles protruding through the sponge surface which appears hispid.

**Figure 8.**
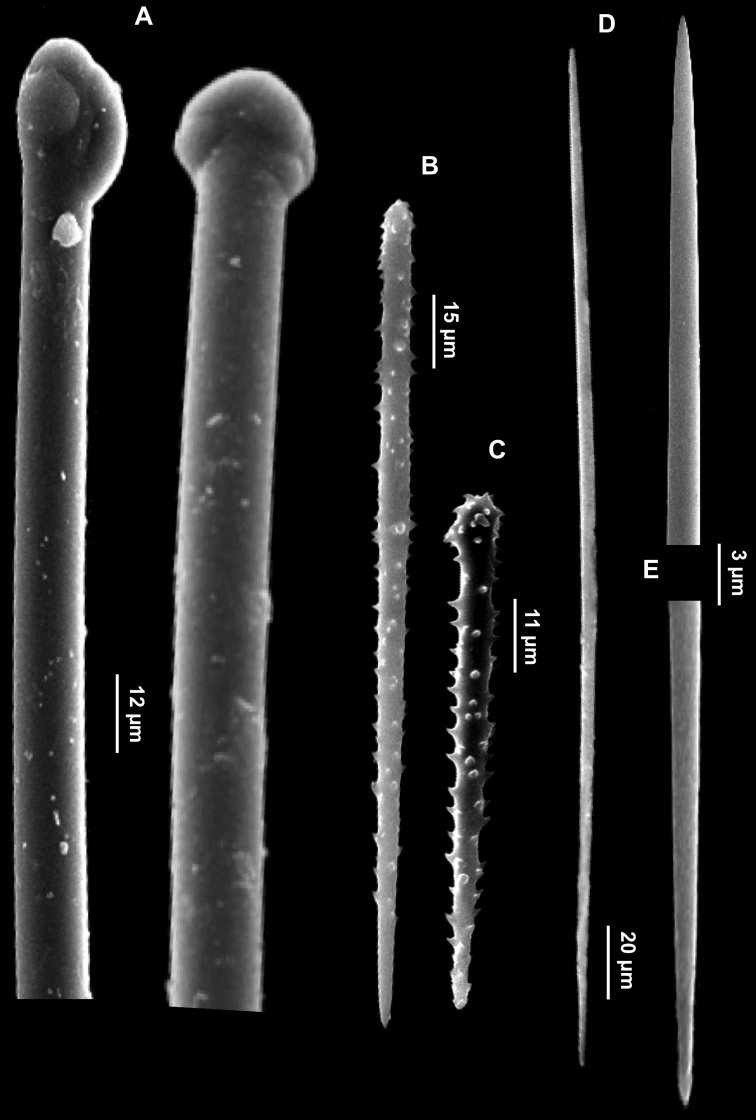
*Eurypon denisae*. **A** Tylostyles with variable head **B** Large acanthostyles **C** Small acanthostyles **D** Anisoxeas **E** Magnifications of the extremities of an anisoxea.

Spicules. Long tylostyles, slightly curved or straight, with rather irregular heads, 1066 (1774) 2236 × 5 (8.5) 12.5 μm (Fig. 8A); anisoxeas straight or faintly curved, 200 (220) 250 × 5 (5.5) 7 μm (Figs 8D-E); acanthostyles in two size categories: I) large, straight acanthostyles, often with inconspicuous heads, uniformly but faintly spined, 107.7 (134.5) 170 × 7.5 (9) 12 μm (Fig. 8B); II) small, straight acanthostyles with stouter and longer spines, 60 (68) 77.5 × 7.5 (8) 10 μm (Fig. 8C).

##### Distribution and discussion.

The species was originally described by Vacelet (1969) from a coral bottom in the bathyal zone (300–350 m depth) of the Gulf of Lions. This second finding is a new record for the Italian seas and the coralligenous community.

#### Eurypon gracilis

Bertolino, Calcinai & Pansini sp. n.

http://zoobank.org/E2792BEE-BEC2-41E5-BB7E-E32969E50A1C

http://species-id.net/wiki/Eurypon_gracilis

Figs 9A–G

##### Material examined.

**Type specimen:** Holotype MSNG 57017. Specimen PdF-S-BL4-sp18-sciaf., on a coralligenous concretion, depth 40 m, Stat. 4, 27-07-2009. leg. M. Bertolino, alcohol preserved.

##### Type locality.

Italy, Ligurian Sea, Portofino Promontory (Punta del Faro) 44°17'54.20"N, 9°13'06.93"E.

##### Other examined material.

Specimen IG-F-BL1-sp4-fot.; specimen IG-F-BL1-sp15-fot.; alcohol preserved, Gallinara Island (station 2, Falconara) 44°01'22"N, 8°13'34"E, depth 35 m, collected 17-06-2009; specimen IG-S-BL3-sp6-fot.; alcohol preserved, Gallinara Island (station 3, Sciusciaù) 44°01'34"N, 8°13'45"E, depth 30 m, collected 17-06-2009; specimen PM-BL1-sp9-sciaf.; alcohol preserved, Punta Manara (station 6) 44°15'05.61"N, 9°24'09.33"E, depth 35 m, collected 13-06-2009.

##### Description.

**Figure 9.**
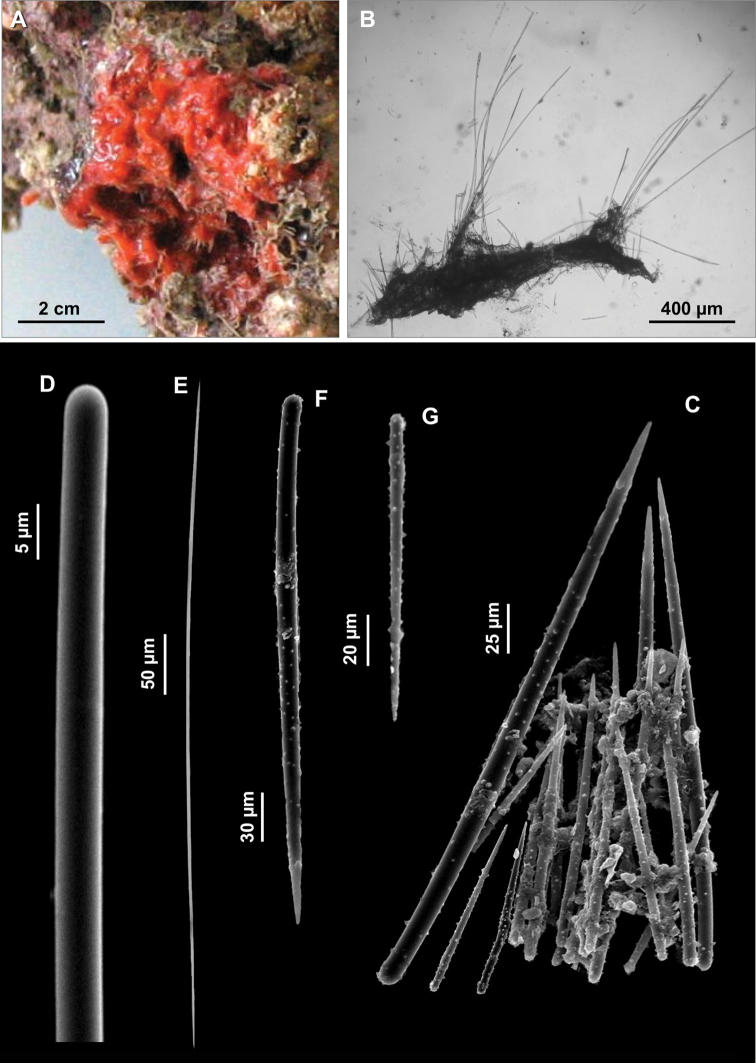
*Eurypon gracilis* sp. n. **A** Holotype **B** Skeleton **C** Portion of the skeleton with large and small echinating acanthostyles **D** Long style **E** Oxea **F** Large acanthostyle with scattered small spines **G** Small acanthostyle.

All the specimens were encrusting on the surface of coralligenous blocks, covering surfaces up to 2 cm^2^. The sponge surface is corrugated, hispid. The colour in life is brick red (Fig. 9A).

Skeleton. The skeleton consists of a basal layer of spongin in which the spicules are vertically positioned, perpendicular to the substrate. Both the categories of acanthostyles are close one another (Fig. 9C) with the heads embedded in the basal spongin layer. Styles and oxeas–with the same vertical arrangement–are grouped in bundles which are faintly echinated, in their lower part, by the smaller acanthostyles (Fig. 9B). Oxeas are positioned in the basal part of the bundles. The styles protrude trough the sponge surface making it hispid.

Spicules. Long styles to tylostyles, curved or flexuous (Fig. 9D), 788 (1101) 1280 × 5 (6.8) 10 µm; oxeas thin, almost straight or with a slight curvature (Fig. 9E), 365 (483) 650 × 2.5 µm; acanthostyles without head and uniformly spined, in two sizes categories: I) large acanthostyles, straight or slightly curved with rather small spines (Fig. 9F), 200 (253) 320 × 5 (6) 7.7 µm; II) small acanthostyles straight, with spines more robust than in the previous category (Fig. 9G), 90 (119.5) 160 × 2.5 (3.8) 5 µm.

##### Etymology.

The species is named after the slenderness of all the spicule types.

##### Distribution.

So far known only from the Ligurian Sea.

##### Ecology.

It lives at 30–40 m depth on coralligenous concretion, characterized by the presence of a *Paramuricea clavata* facies.

##### Discussion.

This species, characterized by a microcionid skeleton with a basal layer of spongin, extra-axial spicules and echinating achantostyles embedded in spongin fibres, clearly belongs to the genus *Eurypon*.

Only five, out of the numerous species of the genus *Eurypon* found in the temperate Western Atlantic have oxeas or tornotes as structural megascleres together with styles or tylostyles. All of them (*Eurypon cinctum* Sarà, 1960, *Eurypon denisae* Vacelet, 1969, *Eurypon obtusum* Vacelet, 1969, *Eurypon major* Sarà & Siribelli, 1960 and *Eurypon lacazei* (Topsent, 1891) occur in the Mediterranean Sea. *Eurypon cinctum* showing a lilac colour, achantostyles with discrete heads and different size in the other megascleres is not close to the new species. *Eurypon denisae* is also different according to the description given above. *Eurypon obtusum* is grey in colour and has smaller oxeas and acanthostyles than those of the present species, but the maximum length of its tylostyles is unknown. *Eurypon lacazei* remarkably differs from the present species for the green colour and spicule shape and size. The closest species to the new one is *Eurypon major* but its tylostyles are longer and stouter (1445–2210 × 10–17 µm) and differ in the shape of the heads, while the acanthostyles, in a single size category, have well formed heads. Only two other species from the temperate Atlantic: *Eurypon lictor* (Topsent, 1904) and *Eurypon (Acantheurypon) mucronale* (Topsent, 1928) present oxeas. However, they are both deep species (recorded deeper than 1500 m from the Azores) and they differ also in the spicule characters from *Eurypon gracilis* sp. n. There are two other species of *Eurypon* with oxeas reported in the literature: *Eurypon calypsoi* Lévi, 1958 from the Red Sea which is blue in colour and *Eurypon fulvum* Lévi, 1969 from South Africa which is yellow. Both have a single size category of acanthostyles and differ in the spicule morphology. *Eurypon gracilis* therefore has to be considered as new for science.

### Suborder Myxillina
Family Coelosphaeridae
Genus *Forcepia*
Subgenus *Leptolabis*

#### Forcepia (Leptolabis) brunnea

(Topsent, 1904)

http://species-id.net/wiki/Forcepia_brunnea

Figs 10A–F

Leptolabis forcipula var. *brunnea* Topsent, 1904: 182.
Leptolabis brunnea Topsent, 1928: 278.

##### Material examined.

Specimen PdF-NE-BL2A-sp15-sciaf.; alcohol preserved, Portofino Promontory (Punta del Faro, station 4) 44°17'55.61"N, 9°13'07.95"E, 40 m depth, collected on 27-08-2009; specimen IG-S-BL3-sp13-sciaf.; alcohol preserved, Gallinara Island (station 3, Sciusciaù) 44°01'34"N, 8°13'45" E, depth 30 m, collected on 17-06-2009; specimen PdF-BL8-sp50-sciaf.; alcohol preserved, Portofino Promontory (Punta del Faro, station 4) 44°17'55.61"N, 9°13'07.95"E, 30 m depth, collected on 25-01-2013.

##### Description.

Thin, smallencrusting sponges (up to 0.5 cm^2^) on the surface of coralligenous blocks. Colour in life yellow-orange.

Skeleton. Basal acanthostyles erect on the substrate in a hymedesmioid arrangement. Other spicule types not detectable from the skeleton.

**Figure 10.**
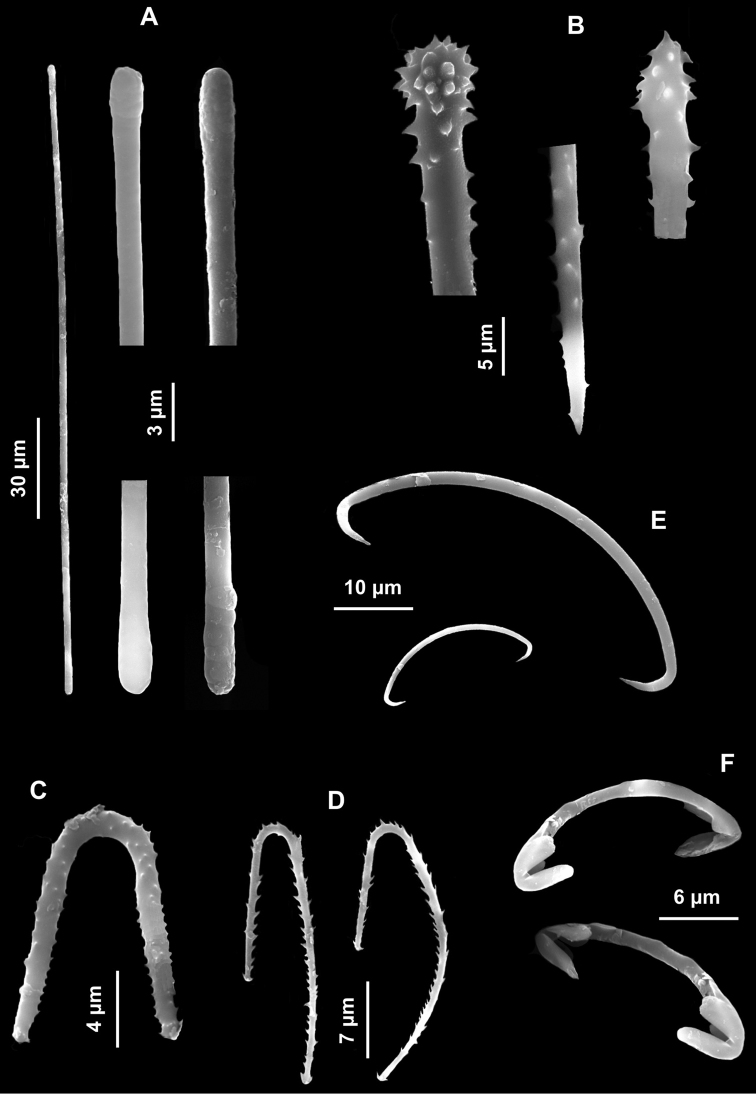
*Forcepia (Leptolabis) brunnea*. **A** Anisotylotes **B** Acanthostyles **C** Symmetric forceps **D** Asymmetric forceps **E** Large and small sigmas **F** Isochelae.

Spicules. Megascleres: anisotylotes straight or faintly curved, with slightly different extremities and a few malformations along the shaft (Fig. 10A), 127.5 (157.7) 280.5 × 1.25 (2.3) 2.5 μm; acanthostyles straight, conical with discrete but not swollen heads. Spines evenly distributed, slightly stouter on the spicule head (Fig. 10B), 61.2 (92.2) 142.8 × 5.2 (7.5) 10.4 μm. Microscleres: acanthose symmetric forceps with straight legs, ending in small, button-like swellings with toothed margin (Fig. 10C). They measure 12.5 (15.8) 17.5 × 2.5 μm in length, the distance between the legs being 5.2 (7.2) 7.5 μm. Acanthose asymmetric forceps, very thin, have unequal legs (Fig. 10D), the longer of which is straight or curved inward, 20.4 (22.3) 25 × 1.5 μm. Sigmas in two size categories: the larger ones, “C" shaped (Fig. 10E) or more rarely “S" shaped, 40.8 (64.3) 80 × 2.5 μm are very abundant, the smaller, 17.5–25.5 μm are rare. Palmate isochelae (Fig. 10F), 18 (20) 20.8 μm long.

##### Distribution and discussion.

Topsent (1904) describes three species of *Leptolabis* from the Azores: *Leptolabis forcipula* var. *brunnea*, *Leptolabis arcuata* and *Leptolabis assimilis*. The same author in 1928 states that the former three species actually belong to a single species: *Leptolabis brunnea* which showsa high variability in the large forceps shape.

*Leptolabis brunnea* was afterwards recorded from the Far-Oer Islands, the Azores, Spain (NW coast, Strait of Gibaltar, Castellón, Girona), France (Marseille, Monaco), Italy (Gulf of Naples), between 4 and 1360 m depth. It lives in caves, detritic bottoms, coralligenous concretions and epibiotic on other organisms (Topsent 1904, 1928, Sarà 1960, Pouliquen 1972, Carballo 1994, Cristobo 1996). This is the second finding for the Italian seas and a new finding for the Ligurian Sea.

### Family Hymedesmiidae
Genus *Hymedesmia*
Subgenus *Hymedesmia*

#### Hymedesmia (Hymedesmia) rissoi

Topsent, 1936

http://species-id.net/wiki/Hymedesmia_rissoi

Figs 11A–D

Hymedesmia gracilisigma var. *rissoi* Topsent, 1936: 35.

##### Material examined.

Specimen IG-F-BL3-F18b-spA; Specimen IG-F-BL4-sp9-sciaf.; specimen IG-F-BL4 sp11-fot.; alcohol preserved, Gallinara Island (station 2, Falconara) 44°01'22"N, 8°13'34"E, depth 35 m, collected on 17-06-2009; specimenSSS-BL1-sp11-sciaf.; Santo Stefano Shoals, (station 1), 43°49'N, 7°54'E, depth 35 m, collected on 14-02-2008.

##### Description.

Small (0.5 cm^2^), slimy, coriaceous encrusting sponge, grey in colour after alcohol preservation, recorded both on the surface and inside the coralligenous blocks.

Skeleton. Not observed.

**Figure 11.**
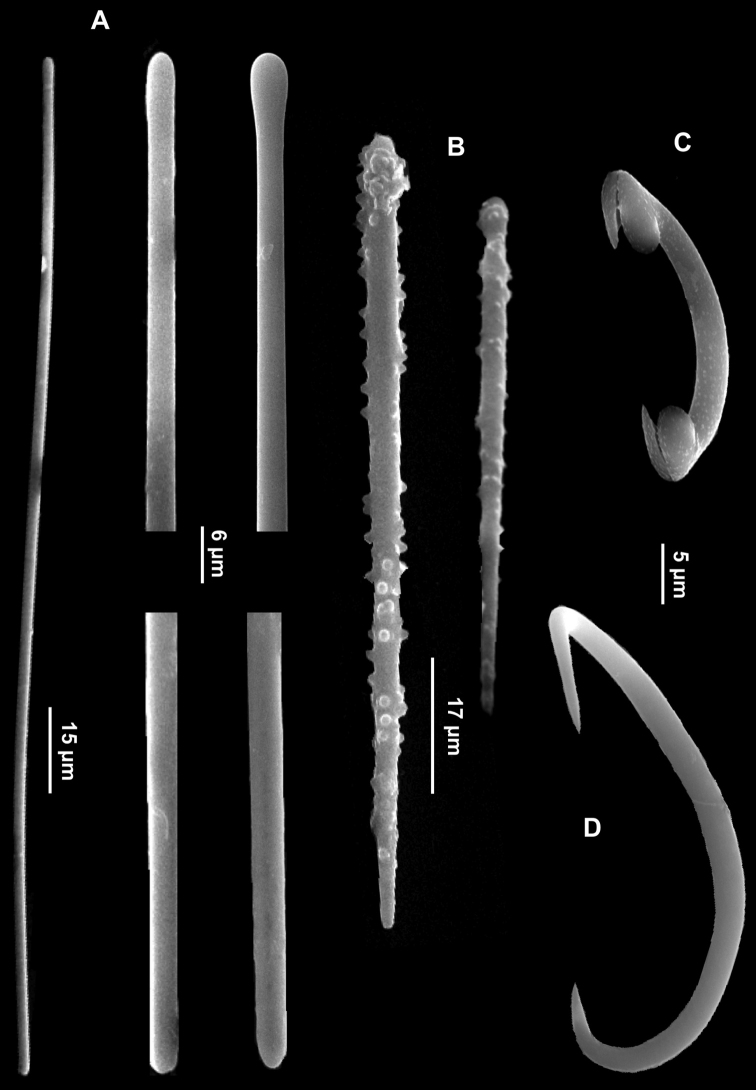
*Hymedesmia (Hymedesmia) rissoi*. **A** Tornote, sometimes modified into subtylotes and strongyles **B** Acanthostyles **C** Arcuate isochelae **D** Thin sigmas.

Spicules. Megascleres: straight or slightly sinuous anisotornotes, sometimes modified in anisotylotes or strongyles (Fig. 11A), 140 (175) 177.5 × 2.5 (2.7) 3.75 μm; acanthostyles in a single size category, 67.5 (84) 105 × 2.5 (3.5) 3.75 μm, devoid of conspicuous heads. The extremities may be pointed or blunt (Figs 11B, C). Microscleres: arcuate isochelae (Fig. 11D), 25 (25.6) 27.5 μm long; thin sigmas “C” (Fig. 11E) and “S” shaped, 32.5 (35) 37.5 × 1.25 μm.

##### Distribution and discussion.

In the original description Topsent (1936) distinguished in this species two size classes of acanthostyles similar in shape: the larger were 185–265 μm in length and the smaller 75–115 μm. Subtylotes straight or sometimes slightly sinuous, 225–275 × 3.5–4.5 μm, arcuate isochelae 23–25 μm long and sigmas 40–50 μm long and less than 1 μm thick. The specimens here described match with Topsent’s description apart from the presence of a single size class of acanthostyles. However, other authors (Sarà and Siribelli 1962), recorded a single class of acanthostyles as well. This is a Mediterranean endemic species (Ligurian Sea and Central Tyrrhenian Sea). It was found on *Cladocora caespitosa*, at 15–40 m depth (Topsent 1936) and on coralligenous bottom, at 40–70 m depth (Sarà and Siribelli 1962).

### Suborder Mycalina
Family Mycalidae
Genus *Mycale*
Subgenus *Paresperella*

#### Mycale (Paresperella) serrulata

Sarà & Siribelli, 1960

http://species-id.net/wiki/Mycale_serrulata

Figs 12A–D

Mycale (Paresperella) serrulata Sarà & Siribelli, 1960: 51.

##### Material examined.

Specimen IG-F-BL3-F4B-spA; specimen IG-F-BL3-F17B-spA alcohol preserved, Gallinara Island (station 2, Falconara) 44°01'22"N, 8°13'34"E, depth 35 m, collected on 31-07-2009. The specimen was entirely used for spicule preparations.

**Description.** Small, encrusting and insinuating sponge, beige in the dry state, occupying a small cavity (1 cm^3^) in a coralligenous block.

Skeleton. Not observed.

**Figure 12.**
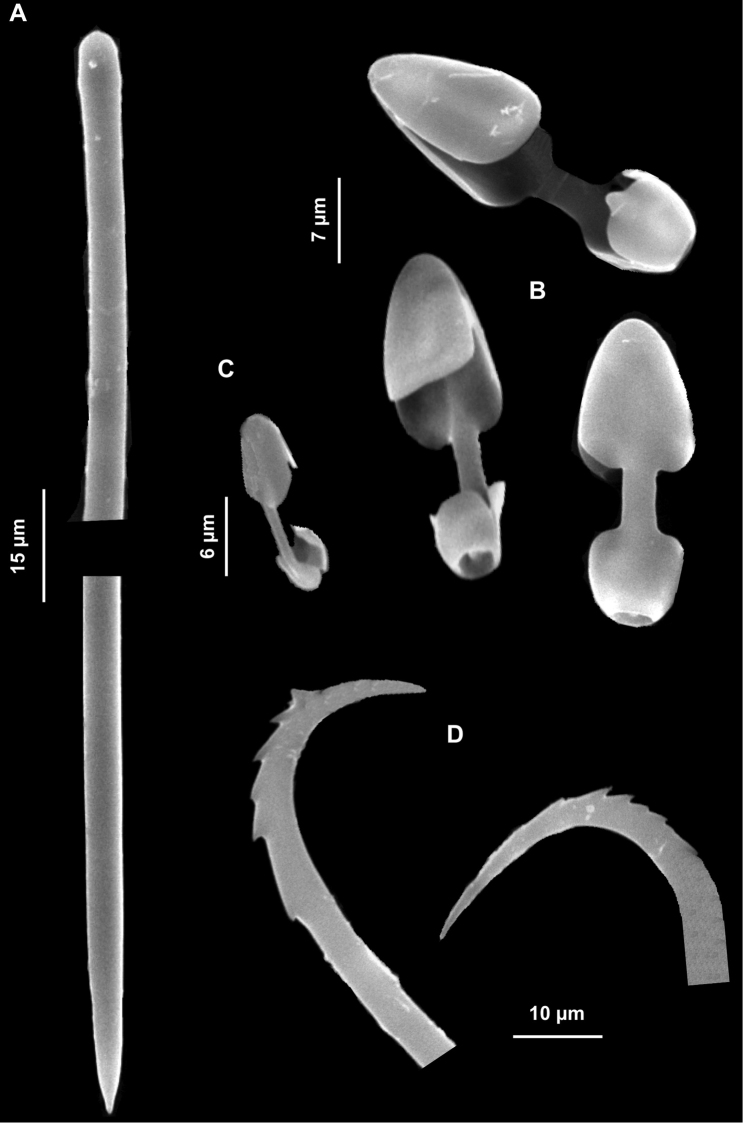
*Mycale (Paresperella) serrulata*. **A–B** Mycalostyles **B** Large anisochelae **C** Small anisochelae **D** Magnifications of the serrated edge of a sigma.

Spicules. Megascleres: mycalostyles straight or flexuous, with acerate tip (Fig. 12A), 310 (325) 340 × 3.75 (5) 7.5 µm. Microscleres: anisochelae in two size categories. I) The larger ones, 25 (29.5) 35 µm, have the bigger tooth palmate and the smaller often characterized by a conspicuous point and slightly diverging outwords alae; a hole is detectable at the smaller extremity (Fig. 12B). II) The smaller ones measure, 12.5 (13.7) 15 µm (Fig. 12C). Sigmas “C” shaped, 64 (78) 100 × 2.5 (2.7) 5 µm, with the convex edge serrated (Fig. 12D).

##### Distribution and discussion.

*Mycale (Paresperella) serrulata* Sarà & Siribelli, 1960, was originally described from a detritic bottom of the Gulf of Naples at 30-40 m depth. Voultsiadou and Vafidis (2004) recorded the species encrusting on *Fasciospongia cavernosa* at 90 m depth in the Aegean Sea. *Mycale (Paresperella) serrulata* is a Mediterranean endemic species. Pansini and Longo (2008) recorded it for the first time for the Ligurian Sea and the coralligenous community.

### Order Halichondrida
Family Eteroxyidae
Genus *Halicnemia*

#### Halicnemia geniculata

Sarà, 1958

http://species-id.net/wiki/Halicnemia_geniculata

Figs 13A–D

Halicnemia geniculata Sarà, 1958: 237.

##### Material examined.

Specimen IG-F-BL4-sp1-sciaf.; alcohol preserved, Gallinara Island (station 2, Falconara) 44°01'22"N, 8°13'34"E, depth 35 m, collected on 17-06-2009. The specimen was entirely used for spicule preparations.

##### Description.

Small and thin, yellow-ochre encrustation (1 cm^2^) on a coralligenous block.

Skeleton. Not observed.

**Figure 13.**
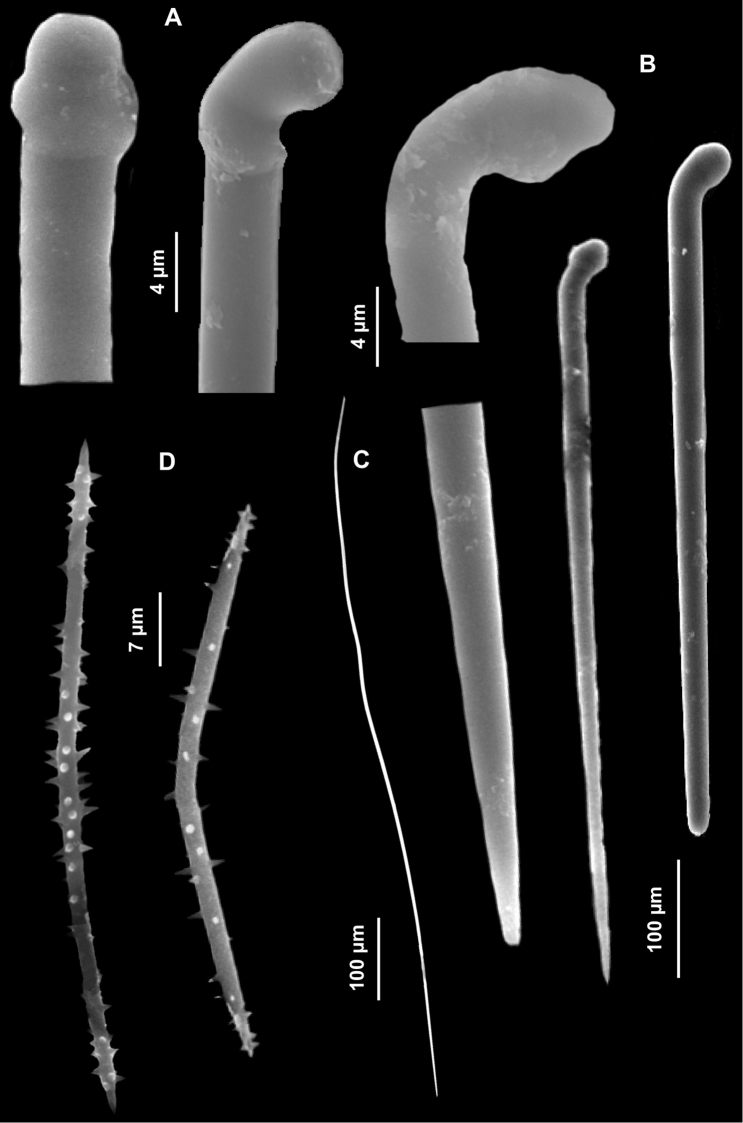
*Halicnemia geniculata*. **A** Magnifications of the tylostyle heads **B** Rabdhotylostyles **C** Oxeas, long, sinuous and thin **D** Acanthoxeas.

Spicules. Long tylostyles, 405 (1351.7) 1976 × 1.5 (2.7) 4 μm, generally straight, with terminal or subterminal swellings variable in shape; irregular and polytylote forms are to be found (Fig. 13A). Rabdhotylostyles with heads as above, 147(242)705 × 1.5 (2.7) 4 μm (Fig. 13B); oxeas long, sinuous and thin, 460 (757) 1118 × 1.5 (2.5) 5 mm (Fig. 13C); acanthoxeas slightly curved or bent, uniformly spined, 42.5 (51.8) 62.5 × 1.5 (1.8) 2 μm (Fig. 13D).

##### Distribution and discussion.

This species, originally described from a superficial cave of the Gulf of Naples (Sarà 1958) was recorded at 60–70 m depth in the same area (Sarà and Siribelli 1962) and in caves close to Marseille (Pouliquen 1972). It is a Mediterranean endemic species (Pansini and Longo 2008) and a new finding for the Ligurian Sea and the coralligenous community.

### Order Haplosclerida
Suborder Haplosclerina
Family Chalinidae
Genus *Haliclona*
Subgenus *Gellius*

#### Haliclona (Gellius) marismedi

(Pulitzer-Finali, 1978)

http://species-id.net/wiki/Haliclona_marismedi

Figs 14A–F

Gellius marismedi , Pulitzer-Finali, 1978: 81.

##### Material examined.

Specimen PM-BL1-sp7-sciaf.; specimen PM-BL1-sp8-sciaf.; specimen PM-BL2b-sp6-sciaf.; specimen PM-BL2b-sp6a-sciaf.; Punta Manara (station 6) 44°15'05.61"N, 9°24'09.33"E, depth 35 m, collected 13-07-2009; specimen IG-S-BL1-sp2-sciaf.; Gallinara Island (station 3, Sciusciaù) 44°01'34"N, 8°13'45"E, depth 30 m, collected on17-06-2009.

**Figure 14.**
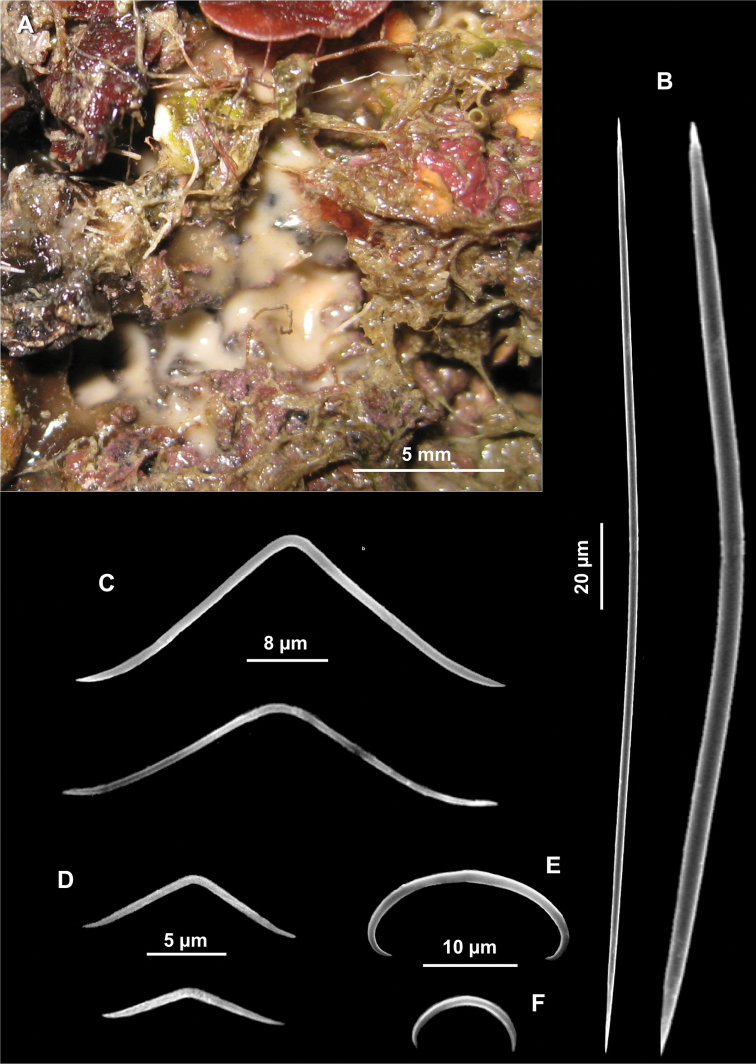
*Haliclona (Gellius) marismedi*. **A** Specimen on the surface of the coralligenous block and insinuating into it **B** Oxeas **C** Large toxas **D** Small toxas **E** Large sigma **F** Small sigma.

**Description.** Small (1-1.5 cm^2^) encrusting and insinuating sponge, beige or brown, detected on the surface and inside a coralligenous block. Surface smooth, consistency soft (Fig. 14A).

Skeleton. The choanosome consists of multispicular primary lines connected by unispicular secondary tracts, creating a confused reticulation.

Spicules. Oxeas gently curved with hastate extremities detectable only in the larger spicules (Fig. 14B), 220 (245) 275 × 2.5 (4.5) 6.25 μm; toxas with more or less angulate central curvature and slightly reflexed points in two size categories: I) 27.5 (45.5) 57.5 μm (Fig. 14C) and II) 10 (11.5) 12.5 μm (Fig. 14D); two types of thin sigmas, “C” shaped, I) 22.5 (23.7) 25 μm and II) 10 (13.6) 17.5 μm (Figs 14E, F).

**Distribution and discussion.** Pulitzer-Finali (1978) described the species from a specimen epibiothic on *Hyrtios collectrix* (Schulze, 1880) found on dead, sanded *Posidonia* beds, at 50 m depth in the Bay of Naples. The same author considered conspecific with *Gellius marismedi* the specimen from Banyuls-sur-Mer (rocky walls in shaded areas at 2–17 m depth and horizontal substrates at 20–40 m depth) attributed to *Gelliodes luridus* (Lundbeck, 1902) by Boury-Esnault (1971).

This is a new finding for the Ligurian Sea and the coralligenous community and the third record after the original description.

## Discussion

According to the latest available revision of coralligenous biodiversity (Ballesteros 2006), 142 species of sponges have been recorded associated with this community. Adding to this list the species recorded on the coralligenous of Apulia (Sarà 1968, 1969), Liguria (Pansini and Pronzato 1973; Calcinai et al. 2007a; Calcinai et al. in prep.; Bertolino et al. 2008) and the Aegean Sea (Kefalas et al. 2003; Kefalas and Castritsi-Catharios 2012) those found associated to red coral (Melone 1965; Templado et al. 1986; Corriero et al. 1988; 1997; Maldonado 1992; Bavestrello et al. 1996; Calcinai et al. 2007b) and the data of the present study, the total number of sponge species hitherto associated to the coralligenous community increases to 273 (Table 2).

**Table 2.** List of sponge species (Demospongiae and Homoscleromorpha) hitherto recorded associated to the coralligenous community.

	**Oscarellidae**
1.	*Oscarella lobularis* (Schmidt, 1862)
	**Plakinidae**
2.	*Corticium candelabrum* Schmidt, 1862
3.	*Placinolopha moncharmonti* (Sarà, 1960)
4.	*Plakina monolopha* Schulze, 1880
5.	*Plakina dilopha* Schulze, 1880
6.	*Plakina trilopha* Schulze, 1880
7.	*Plakinastrella copiosa* Schulze, 1880
8.	*Plakinastrella mixta* Maldonado, 1992
9.	*Plakortis simplex* Schulze, 1880
	**Tetillidae**
10.	*Craniella cranium* (Müller, 1776)
	**Samidae**
11.	*Samus anonymus* Gray, 1867
	**Ancorinidae**
12.	*Stelletta dorsigera* Schmidt, 1862
13.	*Stelletta grubii* Schmidt, 1862
14.	*Stelletta lactea* Carter, 1871
15.	*Stelletta stellata* Topsent, 1893
16.	*Jaspis incrustans* (Topsent, 1890)
17.	*Jaspis johnstonii* (Schmidt, 1862)
18.	*Stryphnus mucronatus* (Schmidt, 1868)
19.	*Stryphnus ponderosus* (Bowerbank, 1866)
20.	*Penares candidata* (Schmidt, 1868)
21.	*Penares euastrum* (Schmidt, 1868)
22.	*Penares helleri* (Schmidt, 1864)
23.	*Holoxea furtiva* Topent, 1892
24.	*Dercitus (Dercitus) bucklandi* (Bowerbank, 1858)
25.	*Dercitus (Stoeba) plicata* (Schmidt, 1868)
	**Calthropellidae**
26.	*Calthropella (Calthropella) pathologica* (Schmidt, 1868)
27.	*Calthropella (Corticellopsis) stelligera* (Schmidt, 1868)
	**Geodiidae**
28.	*Erylus discophorus* (Schmidt, 1862)
29.	*Erylus papulifer* Pulitzer-Finali, 1983
30.	*Caminus vulcani* Schmidt, 1862
31.	*Pachymatisma johnstonia* (Bowerbank in Johnston, 1842)
32.	*Geodia anceps* (Vosmaer, 1894)
33.	*Geodia conchilega* Schmidt, 1862
34.	*Geodia cydonium* Jamenson, 1811
35.	*Caminella intuta* (Topsent, 1892)
	**Pachastrellidae**
36.	*Pachastrella monilifera* Schmidt, 1868
37.	*Poecillastra compressa* (Bowerbank, 1866)
38.	*Nethea amygdaloides* (Carter, 1876)
39.	*Thenea muricata* (Bowerbank, 1858)
40.	*Triptolemma simplex* (Sarà, 1959)
41.	*Vulcanella (Vulcanella) gracilis* (Sollas, 1888)
42.	*Annulastrella verrucolosa* (Pulitzer-Finali, 1983)
	**Clionaidae**
43.	*Cliona burtoni* Topsent, 1932
44.	*Cliona carteri* (Ridley, 1881)
45.	*Cliona celata* Grant, 1826
46.	*Cliona lobata* Hancock, 1849
47.	*Cliona janitrix* Topsent, 1932
48.	*Cliona rhodensis* Rützler & Bromley, 1981
49.	*Cliona schmidtii* (Ridley, 1881)
50.	*Cliona thoosina* Topsent, 1888
51.	*Cliona vermifera* Hancock, 1867
52.	*Cliona viridis* Schmidt, 1862
53.	*Dotona pulchella mediterranea* Rosell & Uriz, 2002
54.	*Pione vastifica* (Hancock, 1849)
55.	*Spiroxya corallophila* (Calcinai, Cerrano & Bavestrello, 2002)
56.	*Spiroxya heteroclita* Topsent, 1896
57.	*Spiroxya levispira* (Topsent, 1898)
58.	*Spiroxya sarai* (Melone, 1965)
	**Thoosidae**
59.	*Alectona millari* Carter, 1879
60.	*Delectona ciconiae* Bavestrello, Calcinai & Sarà, 1996
61.	*Delectona madreporica* Bavestrello et al., 1997
62.	*Thoosa armata* Topsent, 1888
63.	*Thoosa mollis* Volz, 1939
	**Hemiasterellidae**
64.	*Paratimea constellata* (Topsent, 1893)
65.	*Paratimea oxeata* Pulitzer-Finali, 1978
	**Stelligeridae**
66.	*Stelligera rigida* (Montagu, 1818)
	**Polymastiidae**
67.	*Polymastia inflata* Cabioch, 1968
68.	*Polymastia mamillaris* (Müller, 1806)
69.	*Polymastia polytylota* Vacelet, 1969
70.	*Quasillina brevis* (Bowerbank, 1861)
71.	*Pseudotrachya hystrix* (Topsent, 1890)
	**Spirastrellidae**
72.	*Diplastrella bistellata* (Schmidt, 1862)
73.	*Spirastrella cunctatrix* Schmidt, 1868
	**Suberitidae**
74.	*Aaptos aaptos* (Schmidt, 1864)
75.	*Prosuberites longispina* Topsent, 1893
76.	*Protosuberites ectyoninus* (Topsent, 1900)
77.	*Protosuberites epiphytum* (Lamarck, 1815)
78.	*Protosuberites rugosus* (Topsent, 1893)
79.	*Pseudosuberites hyalinus* (Ridley & Dendy, 1867)
80.	*Pseudosuberites sulphureus* (Bowerbank, 1866)
81.	*Suberites carnosus* (Johnston, 1842)
82.	*Suberites carnosus incrustans* Topsent, 1900
83.	*Suberites domuncula* (Olivi, 1792)
84.	*Suberites syringella* (Schmidt, 1868)
85.	*Terpios gelatinosa* (Bowerbank, 1866)
	**Tethyidae**
86.	*Tethya aurantium* (Pallas, 1766)
87.	*Tethya citrina* Sarà & Melone, 1965
	**Timeidae**
88.	*Timea cumana* Pulitzer-Finali, 1978
89.	*Timea fasciata* Topsent, 1934
90.	*Timea irregularis* Sarà & Siribelli, 1960
91.	*Timea stellata* (Bowerbank, 1866)
92.	*Timea stellifasciata* Sarà & Siribelli, 1960
93.	*Timea unistellata* (Topsent, 1892)
	**Trachycladidae**
94.	*Trachycladus minax* (Topsent, 1888)
	**Chondrillidae**
95.	*Chondrosia reniformis* Nardo, 1847
96.	*Chondrilla nucula* Schmidt, 1862
	**Desmanthidae**
97.	*Desmanthus incrustans* (Topsent, 1889)
	**Acarnidae**
98.	*Acarnus souriei* (Lévi, 1952)
99.	*Acarnus tortilis* Topsent, 1892
	**Microcionidae**
100.	*Clathria (Clathria) compressa* (Schmidt, 1862)
101.	*Clathria (Clathria) coralloides* (Olivi, 1792)
102.	*Clathria (Clathria) depressa* Sarà & Melone, 1966
103.	*Clathria (Clathria) toxivaria* (Sarà, 1959)
104.	*Clathria (Microciona) armata* (Bowerbank, 1862)
105.	*Clathria (Microciona) assimilis* Topsent & Olivier, 1943
106.	*Clathria (Microciona) gradalis* Topsent, 1925
107.	*Clathria (Microciona) haplotoxa* (Topsent, 1928)
108.	*Clathria (Microciona) spinarcus* (Carter & Hope, 1889)
109.	*Clathria (Microciona) toxistyla* (Sarà, 1959)
110.	*Antho (Antho) inconstans* (Topsent, 1925)
111.	*Antho (Antho) involvens* (Schmidt, 1864)
112.	*Antho (Acarnia) coriacea* (Bowerbank, 1874)
113.	*Antho (Acarnia)* cf. *novizelanica* (Ridley & Duncan, 1881)
	**Raspailiidae**
114.	*Raspailia (Raspailia) viminalis* Schmidt, 1862
115.	*Aulospongus spinosus* (Topsent, 1927)
116.	*Eurypon cinctum* Sarà, 1960
117.	*Eurypon clavatum* (Bowerbank, 1866)
118.	*Eurypon coronula* (Bowerbank, 1874)
119.	*Eurypon denisae* Vacelet, 1969
120.	*Eurypon gracilis* Present paper
121.	*Eurypon lacazei* (Topsent, 1891)
122.	*Eurypon major* Sarà & Siribelli, 1960
123.	*Eurypon topsenti* Pulitzer-Finali, 1983
124.	*Eurypon vesciculare* Sarà & Siribelli, 1960
125.	*Eurypon viride* (Topsent, 1889)
126.	*Raspaciona aculeata* (Johnston, 1842)
	**Rhabderemiidae**
127.	*Rhabderemia gallica* van Soest & Hooper, 1993
128.	*Rhabderemia indica* Dendy, 1905
129.	*Rhabderemia minutula* (Carter, 1876)
130.	*Rhabderemia* cf. *topsenti* van Soest & Hooper, 1993
	**Chondropsidae**
131.	*Batzella inops* (Topsent, 1891)
	**Coelosphaeridae**
132.	*Chaetodoryx insinuans* (Topsent, 1936)
133.	*Forcepia (Leptolabis) apuliae* (Sarà, 1969)
134.	*Forcepia (Leptolabilis) brunnea* (Topsent, 1904)
135.	*Forcepia (Leptolabis)* cf. *luciensis* (Topsent, 1888)
136.	*Forcepia (Leptolabis) megachela* (Maldonado, 1992)
137.	*Lissodendoryx (Lissodendoryx) isodictyalis* (Carter, 1882)
138.	*Lissodendoryx (Anomodoryx) cavernosa* (Topsent, 1892)
	**Crambeidae**
139.	*Crambe crambe* (Schmidt, 1862)
140.	*Crambe tuberosa* Maldonado & Benito, 1991
	**Crellidae**
141.	*Crella (Crella) elegans* (Schmidt, 1862)
142.	*Crella (Crella) mollior* Topsent, 1925
143.	*Crella (Grayella) pulvinar* (Schmidt, 1868)
144.	*Crella (Pytheas) fusifera* Sarà, 1969
145.	*Crella (Pytheas) sigmata* Topsent, 1925
146.	*Crella (Yvesia) rosea* (Topsent, 1892)
	**Desmacididae**
147.	*Desmacidon adriaticum* Sarà, 1969
148.	*Desmacidon fruticosum* (Montagu, 1818)
	**Hymedesmiidae**
149.	*Hemimycale columella* (Bowerbank, 1864)
150.	*Hymedesmia (Hymedesmia) baculifera* (Topsent, 1901)
151.	*Hymedesmia (Hymedesmia) paupertas* (Bowerbank, 1866)
152.	*Hymedesmia (Hymedesmia) peachi* Bowerbank, 1882
153.	*Hymedesmia (Hymedesmia) plicata* Topsent, 1928
154.	*Hymedesmia (Hymedesmia) rissoi* Topsent, 1936
155.	*Hymedesmia (Hymedesmia) versicolor* (Topsent, 1893)
156.	*Hymedesmia (Stylopus) coriacea* (Fristedt, 1885)
157.	*Phorbas dives* (Topsent, 1891)
158.	*Phorbas fibulatus* (Topsent, 1893)
159.	*Phorbas fictitius* Bowerbank, 1866
160.	*Phorbas mercator* (Schmidt, 1868)
161.	*Phorbas tenacior* (Topsent, 1925)
162.	*Plocamionida ambigua* (Bowerbank, 1866)
	**Myxillidae**
163.	*Myxilla (Myxilla) rosacea* (Lieberkühn,1859)
	**Tedaniidae**
164.	*Tedania (Tedania) anhelans* Lieberkühn, 1849
	**Desmacellidae**
165.	*Biemna parthenopea* Pulitzer-Finali, 1978
166.	*Biemna variantia* (Bowerbank, 1858)
167.	*Desmacella annexa* Schmidt, 1870
168.	*Desmacella inornata* (Bowerbank, 1866)
	**Esperiopsidae**
169.	*Ulosa stuposa* (Esper, 1794)
	**Hamacanthidae**
170.	*Hamacantha (Vomerula) falcula* (Bowerbank, 1874)
	**Mycalidae**
171.	*Mycale (Mycale) lingua* (Bowerbank, 1866)
172.	*Mycale (Mycale) massa* (Schmidt, 1862)
173.	*Mycale (Aegogropila) contarenii* (Lieberkühn, 1859)
174.	*Mycale (Aegogropila) tunicata* (Schmidt, 1862)
175.	*Mycale (Paresperella) serrulata* Sarà & Siribelli, 1960
	**Merliidae**
176.	*Merlia normani* Kirkpatrick, 1908
	**Podospongiidae**
177.	*Podospongia lovenii* Bocage, 1870
	**Latrunculiidae**
178.	*Latrunculia (Biannulata) citharistae* Vacelet, 1969
179.	*Sceptrella biannulata* (Topsent, 1892)
180.	*Sceptrella insignis* (Topsent, 1890)
	**Axinellidae**
181.	*Axinella cannabina* (Esper, 1794)
182.	*Axinella damicornis* (Esper, 1794)
183.	*Axinella rugosa* (Bowerbank, 1866)
184.	*Axinella polypoides* Schmidt, 1862
185.	*Axinella verrucosa* (Esper, 1794)
186.	*Phakellia robusta* Bowerbank, 1866
187.	*Phakellia ventilabrum* (Linnaeus, 1767)
	**Bubaridae**
188.	*Bubaris carcisis* Vacelet, 1969
189.	*Bubaris vermiculata* (Bowerbank, 1866)
190.	*Cerbaris curvispiculifer* (Carter, 1880)
191.	*Monocrepidion vermiculatum* Topsent, 1898
	**Hymerhabdiidae**
192.	*Hymerhabdia oxytrunca* Topsent, 1904
193.	*Hymerhabdia typica* Topsent, 1892
	**Heteroxyidae**
194.	*Halicnemia geniculata* Sarà, 1958
195.	*Halicnemia patera* Bowerbank, 1864
	**Dictyonellidae**
196.	*Acanthella acuta* Schmidt, 1862
197.	*Dictyonella incisa* (Schmidt, 1880)
198.	*Dictyonella marsilii* (Topsent, 1893)
199.	*Dictyonella obtusa* (Schmidt, 1862)
200.	*Dictyonella pelligera* (Schmidt, 1862)
	**Halichondriidae**
201.	*Axinyssa aurantiaca* (Schmidt,1864)
202.	*Halichondria (Halichondria) bowerbanki* Burton, 1930
203.	*Halichondria (Halichondria) contorta* (Sarà, 1961)
204.	*Halichondria (Halichondria) convolvens* Sarà, 1960
205.	*Halichondria (Halichondria) genitrix* (Schmidt, 1870)
206.	*Halichondria (Halichondria) panicea* (Pallas, 1766)
207.	*Halichondria (Halichondria) semitubulosa* Lieberkühn, 1859
208.	*Hymeniacidon perlevis* (Montagu, 1818)
209.	*Hymeniacidon rugosa* (Schmidt, 1868)
210.	*Laminospongia subtilis* Pulitzer-Finali, 1983
211.	*Spongosorites intricatus* (Topsent, 1892)
212.	*Spongosorites flavens* Pulitzer-Finali, 1983
213.	*Topsentia glabra* (Topsent, 1898)
214.	*Topsentia vaceleti* Kefalas & Castritsi–Catharios, 2012
	**Agelasidae**
215.	*Agelas oroides* Schmidt, 1864
	**Callyspongiidae**
216.	*Callyspongia subcornea* Griessinger, 1971
	**Chalinidae**
217.	*Dendroxea lenis* (Topsent, 1892)
218.	*Haliclona (Gellius) angulata* (Bowerbank, 1866)
219.	*Haliclona (Gellius) dubia* (Babic, 1922)
220.	*Haliclona (Gellius) flagellifer* (Ridley & Dendy, 1866)
221.	*Haliclona (Gellius) lacazei* (Topsent, 1893)
222.	*Haliclona (Gellius) marismedi* (Pulitzer-Finali, 1978)
223.	*Haliclona (Gellius) tenuisigma* (Sarà & Siribelli, 1960)
224.	*Haliclona (Halichoclona) fulva* (Topsent, 1893)
225.	*Haliclona (Haliclona) simulans* (Johnston, 1842)
226.	*Haliclona (Reniera) aquaeductus* (Schmidt, 1862)
227.	*Haliclona (Reniera) citrina* (Topsent, 1892)
228.	*Haliclona (Reniera) cratera* (Schmidt, 1862)
229.	*Haliclona (Reniera) mediterranea* Griessinger, 1971
230.	*Haliclona (Rhizoniera) rosea* (Bowerbank, 1866)
231.	*Haliclona (Rhizoniera) sarai* (Pulitzer-Finali, 1969)
232.	*Haliclona (Soestella) arenata* Griessinger, 1971
233.	*Haliclona (Soestella) implexa* (Schmidt, 1868)
234.	*Haliclona (Soestella) mamillata* (Griessinger, 1971)
235.	*Haliclona (Soestella) mucosa* (Griessinger, 1971)
236.	*Haliclona (Soestella) valliculata* (Griessinger, 1971)
237.	*Haliclona elegans* (Lendenfeld, 1887)
	**Phloeodictyidae**
238.	*Siphonodictyon coralliirubri* (Calcinai et al., 2007)
239.	*Siphonodictyon insidiosum* (Johnson, 1899)
240.	*Calyx nicaeensis* (Risso, 1826)
	**Petrosiidae**
241.	*Petrosia (Petrosia) clavata* (Esper, 1794)
242.	*Petrosia (Petrosia) ficiformis* (Poiret, 1798)
	**Irciniidae**
243.	*Ircinia dendroides* (Schmidt, 1862)
244.	*Ircinia oros* (Schmidt, 1864)
245.	*Ircinia variabilis* (Pallas, 1766)
246.	*Sarcotragus fasciculatus* (Pallas, 1766)
247.	*Sarcotragus foetidus* Schmidt, 1862
248.	*Sarcotragus pipetta* (Schmidt, 1868)
249.	*Sarcotragus spinosulus* Schmidt, 1862
	**Thorectidae**
250.	*Cacospongia mollior* Schmidt, 1862
251.	*Cacospongia scalaris* Schmidt, 1862
252.	*Hyrtios collectrix* (Schulze, 1880)
253.	*Fasciospongia cavernosa* (Schmidt, 1862)
	**Spongiidae**
254.	*Spongia (Spongia) agaricina* Pallas, 1766
255.	*Spongia (Spongia) nitens* (Schmidt, 1862)
256.	*Spongia (Spongia) officinalis* Linnaeus, 1759
257.	*Spongia (Spongia) virgultosa* (Schmidt, 1868)
258.	*Spongia (Spongia) zimocca* Schmidt, 1862
259.	*Hippospongia communis* (Lamarck, 1814)
	**Dysideidae**
260.	*Dysidea avara* (Schmidt, 1862)
261.	*Dysidea fragilis* (Montagu, 1818)
262.	*Dysidea tupha* (Martens, 1824)
263.	*Pleraplysilla spinifera* (Schulze, 1879)
	**Darwinellidae**
264.	*Aplysilla rosea* (Barrois, 1876)
265.	*Aplysilla sulfurea* Schulze, 1878
266.	*Chelonaplysilla noevus* (Carter, 1876)
	**Dictyodendrillidae**
267.	*Spongionella gracilis* (Vosmaer, 1883)
268.	*Spongionella pulchella* (Sowerby, 1804)
	**Halisarcidae**
269.	*Halisarca dujardini* Johnston, 1842
	**Aplysinidae**
270.	*Aplysina aerophoba* Nardo, 1843
271.	*Aplysina cavernicola* Vacelet, 1959
	**Ianthellidae**
272.	*Hexadella pruvoti* Topsent, 1896
273.	*Hexadella racovitzai* Topsent, 1896

This increasing is related to the difficulty of studying the organisms inhabiting the coralligenous concretions due to the complexity of the habitat, the high diversity, and the depth where these structures are located (Kipson et al. 2011). Our study, based on the collection of blocks and their sectioning into slices, allowed the identification of species that would have been otherwise completely disregarded.

Among the insinuating species observed in the coralligenous crevices we have found several species previously recorded with a massive habitus in deeper waters. *Pachastrella monilifera* Schmidt, 1868 and *Poecillastra compressa* (Bowerbank, 1866) were the species with the highest phenotypic plasticity, since they usually appear with large, fun shaped specimens, in deep habitats (Bo et al. 2012), while in the coralligenous community they live in crevices and fissures of the concretion. Our results support the idea that environments rich in microhabitats may act as shelters essential for the dispersal of many deep water species, enlarging their distribution range (Bo et al. 2011). Therefore we can emphasize the importance of the coralligenous concretion, not only as reservoir of biodiversity, but also as an important “stepping-stone” able to facilitate the dispersal of species along vertical gradients.

As to the boring sponges, *Cliona janitrix* is indicated by Ballesteros (2006) and Calcinai et al. (2007b) as the key species in the bio-erosive processes involving *Corallium rubrum*, whereas *Cliona viridis* has the same rolein the coralligenous matrix (Rosell et al. 1999). According to our data *Cliona celata* Grant, 1826, *Cliona schmidtii* (Ridley, 1881), *Spiroxya corallophila* (Calcinai, Cerrano & Bavestrello, 2002), *Spiroxya heteroclita* Topsent, 1896 and *Siphonodictyon insidiosum* (Johnson, 1899) may also be considered important in the bio erosive processes acting upon the coralligenous structure. SEM analyses showed that three other species: *Jaspis johnstoni* (Schmidt, 1862), *Dercitus (Stoeba) plicatus* (Schmidt, 1868), *Samus anonymus* Gray, 1867, suspected to be excavating (Carter 1880, Thomas 1973, van Soest and Hooper 2002), actually do not bore the coralligenous substratum but only occupy cavities of the porous concretion and the chambers previously excavated by boring sponges (Figs 2E–F). *Cliona viridis*, *Jaspis johnstoni* and *Dercitus (Stoeba) plicatus*, able to penetrate 5 cm into the substrate, are the species reaching the greatest depth inside the concretion.

## Supplementary Material

XML Treatment for Cliona burtoni

XML Treatment for Paratimea oxeata

XML Treatment for Clathria (Microciona) armata

XML Treatment for Clathria (Microciona) haplotoxa

XML Treatment for Eurypon denisae

XML Treatment for Eurypon gracilis

XML Treatment for Forcepia (Leptolabis) brunnea

XML Treatment for Hymedesmia (Hymedesmia) rissoi

XML Treatment for Mycale (Paresperella) serrulata

XML Treatment for Halicnemia geniculata

XML Treatment for Haliclona (Gellius) marismedi
